# Molecular dynamics analysis of N-acetyl-D-glucosamine against specific SARS-CoV-2’s pathogenicity factors

**DOI:** 10.1371/journal.pone.0252571

**Published:** 2021-05-27

**Authors:** Ömür Baysal, Naeem Abdul Ghafoor, Ragıp Soner Silme, Alexander N. Ignatov, Volha Kniazeva

**Affiliations:** 1 Faculty of Science, Department of Molecular Biology and Genetics, Molecular Microbiology Unit, Muğla Sıtkı Koçman University, Menteşe-Muğla, Turkey; 2 Center for Research and Practice in Biotechnology and Genetic Engineering, Istanbul University, Istanbul, Turkey; 3 Federal State Autonomous Educational Institution, People’s Friendship University of Russia, Moscow, Russia; 4 Institute of Biophysics and Cell Engineering of the National Academy of Sciences of Belarus, Minsk, Belarus; Dokuz Eylul Universitesi, TURKEY

## Abstract

The causative agent of the pandemic identified as SARS-CoV-2 leads to a severe respiratory illness similar to SARS and MERS with fever, cough, and shortness of breath symptoms and severe cases that can often be fatal. In our study, we report our findings based on molecular docking analysis which could be the new effective way for controlling the SARS-CoV-2 virus and additionally, another manipulative possibilities involving the mimicking of immune system as occurred during the bacterial cell recognition system. For this purpose, we performed molecular docking using computational biology techniques on several SARS-CoV-2 proteins that are responsible for its pathogenicity against N-acetyl-D-glucosamine. A similar molecular dynamics analysis has been carried out on both SARS-CoV-2 and anti-*Staphylococcus aureus* neutralizing antibodies to establish the potential of N-acetyl-D-glucosamine which likely induces the immune response against the virus. The results of molecular dynamic analysis have confirmed that SARS-CoV-2 spike receptor-binding domain (PDB: 6M0J), RNA-binding domain of nucleocapsid phosphoprotein (PDB: 6WKP), refusion SARS-CoV-2 S ectodomain trimer (PDB: 6X79), and main protease 3clpro at room temperature (PDB: 7JVZ) could bind with N-acetyl-D-glucosamine that these proteins play an important role in SARS-CoV-2’s infection and evade the immune system. Moreover, our molecular docking analysis has supported a strong protein-ligand interaction of N-acetyl-D-glucosamine with these selected proteins. Furthermore, computational analysis against the D614G mutant of the virus has shown that N-acetyl-D-glucosamine affinity and its binding potential were not affected by the mutations occurring in the virus’ receptor binding domain. The analysis on the affinity of N-acetyl-D-glucosamine towards human antibodies has shown that it could potentially bind to both SARS-CoV-2 proteins and antibodies based on our predictive modelling work. Our results confirmed that N-acetyl-D-glucosamine holds the potential to inhibit several SARS-CoV-2 proteins as well as induce an immune response against the virus in the host.

## Introduction

The Severe Acute Respiratory Syndrome Coronavirus 2 (SARS-CoV-2) has led to the pandemic cases with significant rises in number of patients in the world. The main theory supported by scientific evidence was the outbreak started from a local seafood market in Huanan showed that human-to-human transmission of the virus was not limited [1]. Globally, pandemic has already caused more than 160 million confirmed cases, in nearly 210 other countries (including around 5 million cases in Turkey, 4.9 million cases in Russia and 371.000 cases in the Republic of Belarus), which has become a unique public health event attracting public attention [2].

SARS-CoV-2 is a positive-chain RNA virus that belongs to the beta group of coronaviruses. The SARS-CoV-2 genome consists of approximately 29.700 nucleotides and has 79.5% sequence identity with SARS-CoV. It has a long polyprotein ORF1ab at the 5’ end, which encodes 15 or 16 non-structural proteins. The 3’ end of the genome encodes 4 major structural proteins, including the spike protein (S), the nucleocapsid protein (N), the membrane protein (M), and the envelope protein (E) [3,4]. SARS-CoV-2 binds to the receptor of angiotensin-converting enzyme 2 (ACE2) on the host cell for virus penetration and subsequent pathogenesis, leading to severe respiratory disease with symptoms of fever, cough, shortness of breath and severe cases that could be fatal [5,6].

Coronaviruses have error-prone RNA-dependent RNA polymerases, mutations, and recombination events occurring frequently that raise the concern regarding its rapidly strengthening and its increased capacity to cause disease [7]. Many mutations detected on the virus genome suggest the formation of the strains with high pathogenicity and contagiousness, which makes the control of the pathogen quite difficult [8]. In our previous study, we have investigated unvarying regions with less mutations than the other parts of SARS-CoV-2 genome obtained from 134 different genome sequences of the GISAID database from distinct parts of the world. The amino acid sequence of the conserved region (ORF1ab region) was obtained, then subjected to homology modeling and introduced N-acetyl-D-glucosamine (D-GlcNAc) as a potential inhibitor for the selected proteins; spike receptor-binding domain bound with ACE2 (PDB 6M0J) and RNA-binding domain of nucleocapsid phosphoprotein (PDB 6WKP) from SARS-CoV-2 [9]. Previous studies have also indicated that the effectiveness of D-GlcNAc against influenza and as a starting material of oseltamivir, a purposed drug for SARS-Cov-2 treatment, D-GlcNAc has been transformed into azido group followed by implantation of a 3-pentoxy group of the desired chemical structure [10,11]. While, oseltamivir has very complex structure which causes side effects involving nausea, vomiting, headaches, kidney, and psychiatric events [12]. In another study, Song and colleagues reported that the O-GlcNAcylation of mitochondrial antiviral-signaling protein (MAVS) which is a key mediator of interferon signaling that plays role in regulation to activate the host innate immunity against RNA viruses [13]. Accordingly, a previous research demonstrated that, azithromycin can be combined with glucosamine early in the course of RNA virus infections, which could aid the control of enhanced type 1 interferon induction [14]. Currently, there are some specific antiviral vaccines or therapies to treat SARS-CoV-2, but their long-lasting effects remain unclear. We should also consider drug repurposing due to its beneficial properties for searching new advantages or purposes of the existing drugs that could reduce the cost and time of drug development and lower the risk of unexpected side effects of the drug [15,16].

In this present study, we focused on the interaction of four different proteins playing major role in SARS-CoV-2 pathogenesis with D-GlcNAc by using molecular docking and further validating the results with molecular dynamics [9].

## Materials and methods

### Retrieving the protein structures

**Fig 1:**
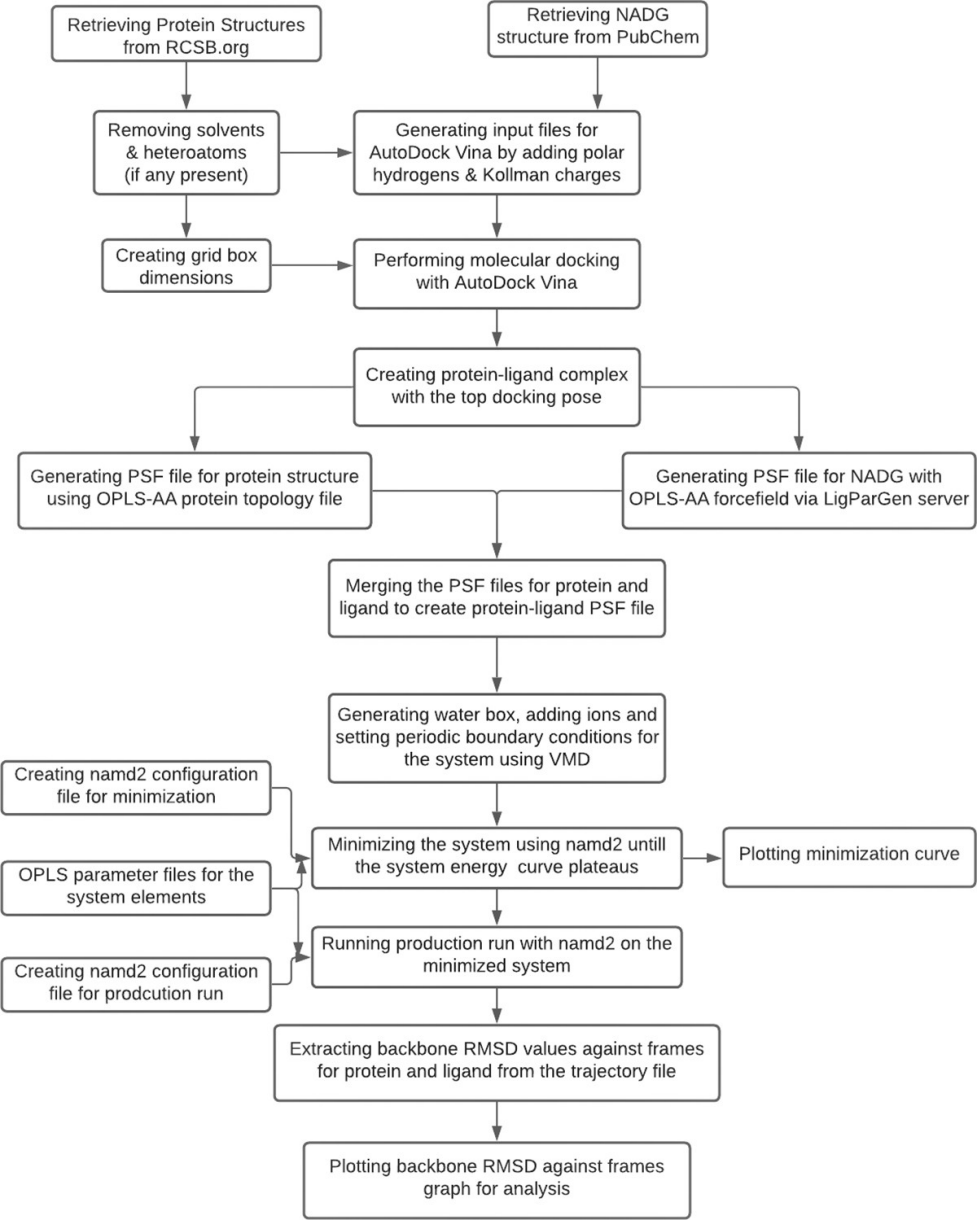
General workflow applied for all the proteins analyzed in the study.

We retrieved the crystal structure of SARS-CoV-2 spike receptor-binding domain (PDB: 6M0J), crystal structure of RNA-binding domain of nucleocapsid phosphoprotein from SARS-CoV-2 monoclinic crystal form (PDB: 6WKP), electron microscopy structure of refusion SARS-CoV-2 S ectodomain trimer covalently stabilized in the closed conformation (PDB: 6X79), and X-ray diffraction structure of SARS-CoV-2 main protease 3clpro (Mpro) at room temperature (damage-free XFEL monoclinic, PDB: 7JVZ) from RCSB website. The structure of the ligand D-GlcNAc was retrieved from PubChem (CID: 439174) [17–21]. The general workflow applied to each protein analyzed is shown in Fig 1.

### Preparation of structure files for molecular docking analysis

As for structure preparations, all of the four protein PDB files had their water and heteroatoms (except for ions) removed using UCSF Chimera, then they were loaded into AutoDock 4.2 where only polar hydrogen atoms and Kollman charges were added, the structures were then exported as PDBQT files. As for the ligand, it was prepared using a similar procedure with further geometrical optimization using the MMFF94 forcefield [22–24].

### Molecular docking analysis

Docking experiments were performed using AutoDock Vina on a Linux cluster [25]. Docking of 6M0J and 6WKP were performed with exhaustiveness of 64 and 24 modes, the grid box calculated for each were 25 x 50 x 20 Å from their center (-12.972, 19.833, 0.667) and 14 x 16 x 12 Å from their center (4.36, -5.444, 22.056) along their X, Y, and Z axes, respectively. Blind docking experiments were performed for 6X79 and 7JVZ with exhaustiveness of 128 and 32 modes, the grid box calculated for each were 122.456 x 130.663 x 149.16 Å and 40.222 x 63.997 x 61.273 Å from the center of the protein along their X, Y, and Z axes, respectively. The best docking pose with the highest affinity (lowest kcal/ mol) was selected.

### Preparation of structure files for molecular dynamics analysis

The best docking pose for each structure was produced using UCSF Chimera’s ViewDock plugin and later the protein and ligand (D-GlcNAc) were saved in separate files. The ligands poses were submitted to LigParGen for the generation of topology and parameter files using OPLS-AA forcefield [26–28]. The protein structure files for each protein were generated using OPLS-AA/M topology for proteins in VMD using the psfgen builder plugin [27,29]. The protein structure file (PSF) and the protein data bank file (PDB) for each protein was merged with its corresponding ligand PSF and PDB files (from LigParGen) into a single protein-ligand complex PSF and PDB files. Each protein-ligand complex was solvated with water in a rectangular box, the box dimensions were determined such that every edge was 5 Å away from the complex. The box was further neutralized with 0.15 mol/ L NaCl such that a distance of 5 Å was maintained between the ions and the solute, and between the ions themselves.

### Molecular dynamics analysis

Molecular dynamics simulations were performed using the University of Illinois Nanoscale Molecular Dynamics (NAMD) software [30]. Each simulation was run using the solvated and ionized protein-ligand complex with OPLS-AA/M protein parameter files and their respective D-GlcNAc parameter files from the LigParGen server, as for the water and ions, the topology file from CHARMM-GUI was utilized [31]. All the protein-ligand complexes were minimized (energy minimization) with the steepest descent algorithm under NVT ensemble at 310 K for 50.000 steps except 6M0J which was minimized with NVT ensemble at 310 K for 100.000 steps, the graphs of backbone Root-mean-square deviation (RMSD) against the frames were plotted to check if the systems were successfully minimized. After confirming the minimization, each protein-ligand complex was simulated for 1.000.000 steps (≈1 ns), each time step was set to 1 fs and the outputs were written to the trajectory every 50 steps, the simulations were run under Periodic Boundary Conditions (PBC) with space partitioning cutoff set to 10, initial and bath temperature set to 310 K with Langevin dynamics at the same temperature. All the water molecules and the protein-ligand complex were wrapped around the PBC. The graph of backbone RMSD against the frames were plotted for each simulation to analyze the protein-ligand complexes’ stability.

### D-GlcNAc-antibody affinity analysis

Two neutralizing antibodies, one specific to the SARS-CoV-2 spike protein from PDB: 7JV2 and another anti-*Staphylococcus aureus* from PDB: 6P9H were extracted from their structures via UCSF Chimera, the structures were prepared for docking with the same configurations in the previous step, and blind docking was performed via AutoDock Vina. The top pose for each antibody-D-GlcNAc was simulated via NAMD with the same procedures and configurations as the previous molecular dynamic simulation. The backbone RMSD of the antibodies and D-GlcNAc was plotted for further analysis.

## Results

### Molecular docking

**Fig 2:**
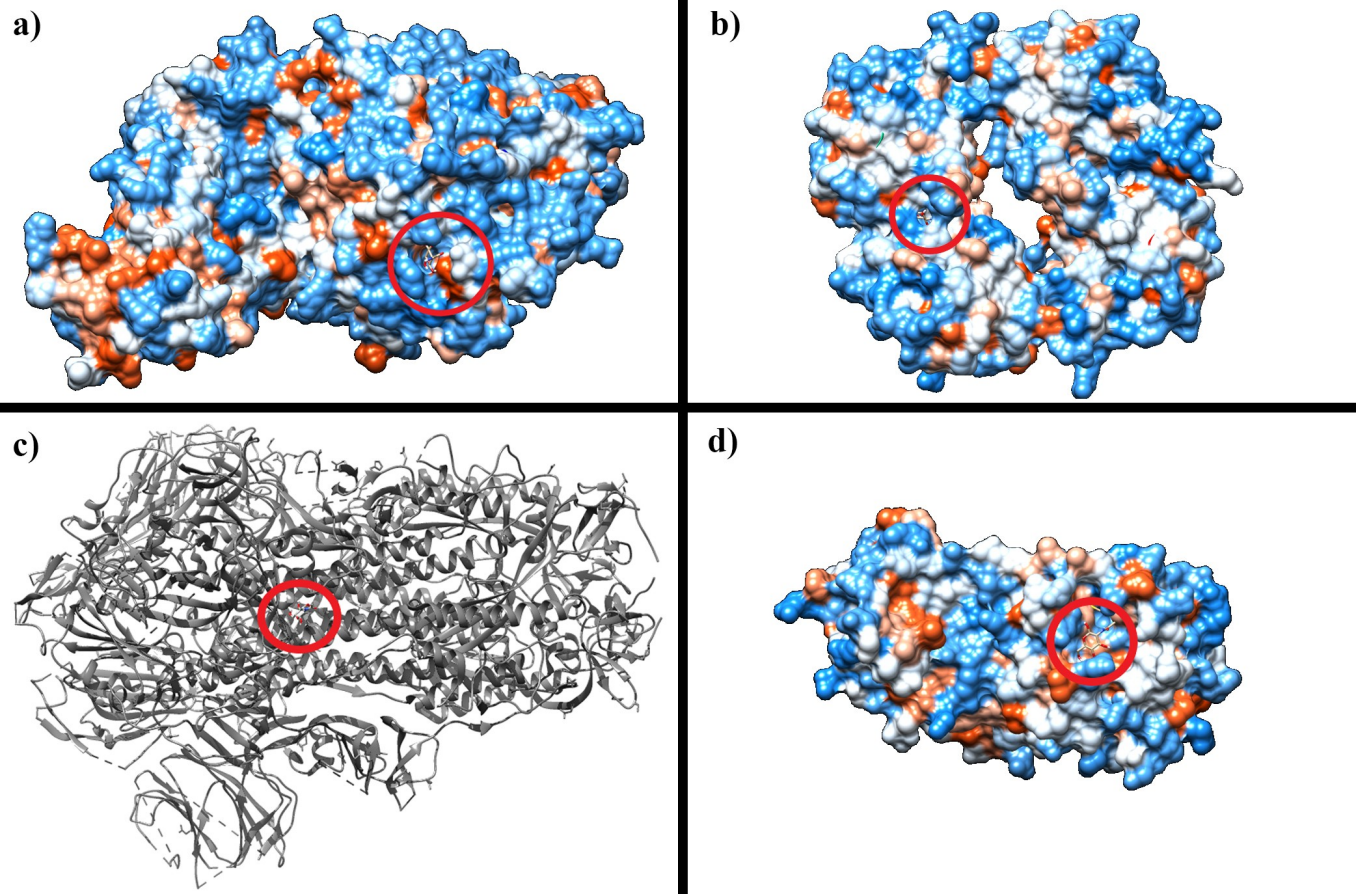
Best docking poses for tested proteins. Hydrophobicity surface representation of the best docking pose with D-GlcNAc for **a**) 6M0J, **b**) 6WKP, **d**) 7JVZ and ribbon representation to the best docking pose with D-GlcNAc for **c**) 6X79. Red circles showing where D-GlcNAc is positioned within the protein. Figures were produced with UCSF Chimera.

All the results are promising as D-GlcNAc has shown a decent affinity to each of the protein structure. Table 1 shows the results of the best-docked pose in the molecular docking experiment using AutoDock Vina. The docking poses corresponding to the values in Table 1 are represented with hydrophobicity surface representation for 6M0J (A), 6WKP (B), and 7JVZ (D) and with ribbon representation for 6X79 (C) using UCSF Chimera in Fig 2 with red circles showing D-GlcNAc position. The log files showing all the poses generated by AutoDock Vina are included in S1 Data.

**Table 1:** Docking pose of D-GlcNAc with the highest affinity (lowest kcal/ mol) to each of the tested proteins from AutoDock Vina.

Docked structure	Affinity (kcal/ mol)
6M0J	-5.1
6WKP	-6.6
6X79	-6.6
7JVZ	-5.6

**Fig 3:**
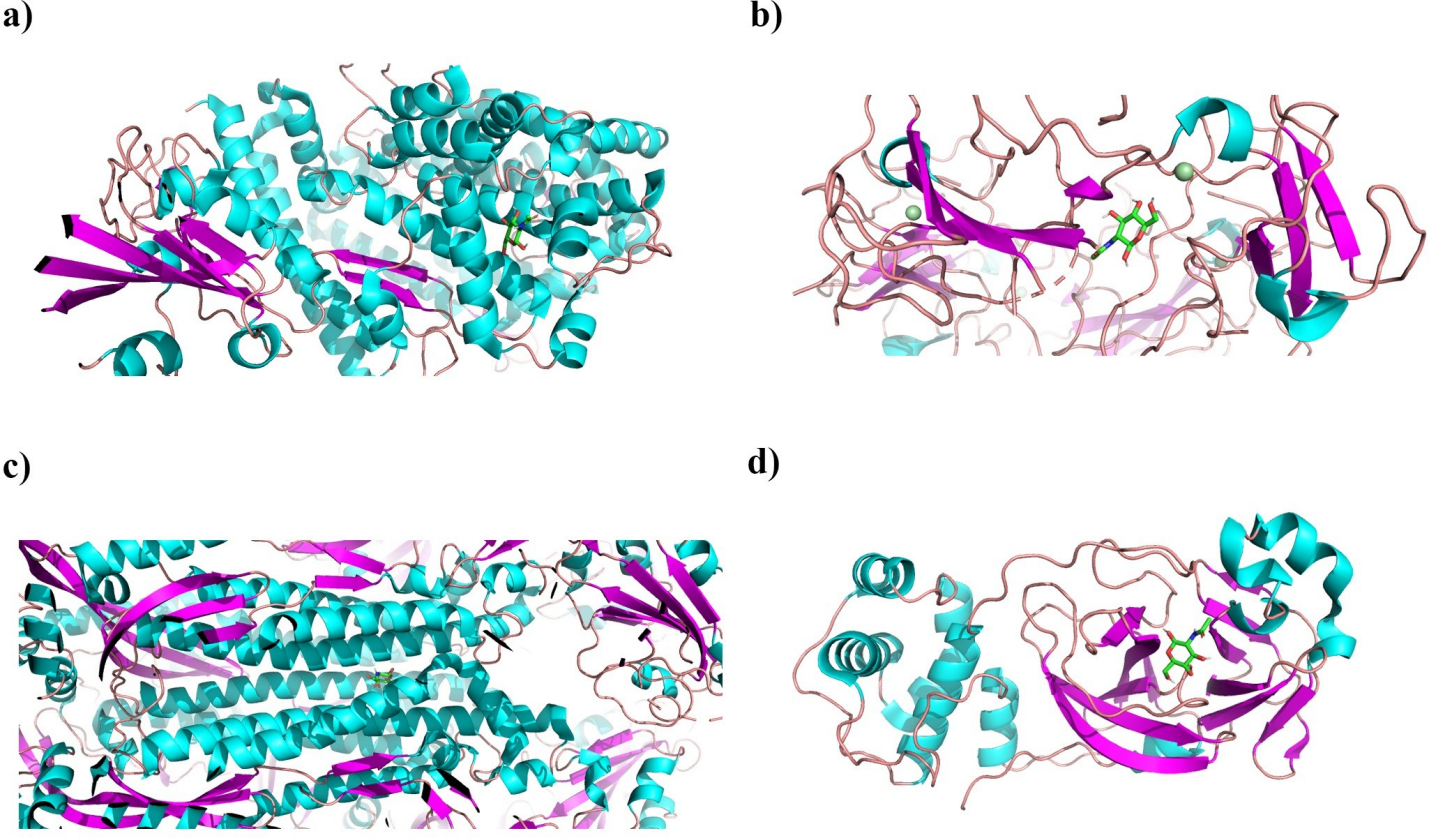
Best docking poses for tested proteins. Cartoon representation of the best docking pose of proteins (colored by secondary structures, clipped for visibility of the ligand) with D-GlcNAc for **a**) 6M0J, **b**) 6WKP, **d**) 7JVZ, and ribbon representation to the best docking pose with D-GlcNAc for **c**) 6X79. Figures were produced with PyMol [32].

**Fig 4:**
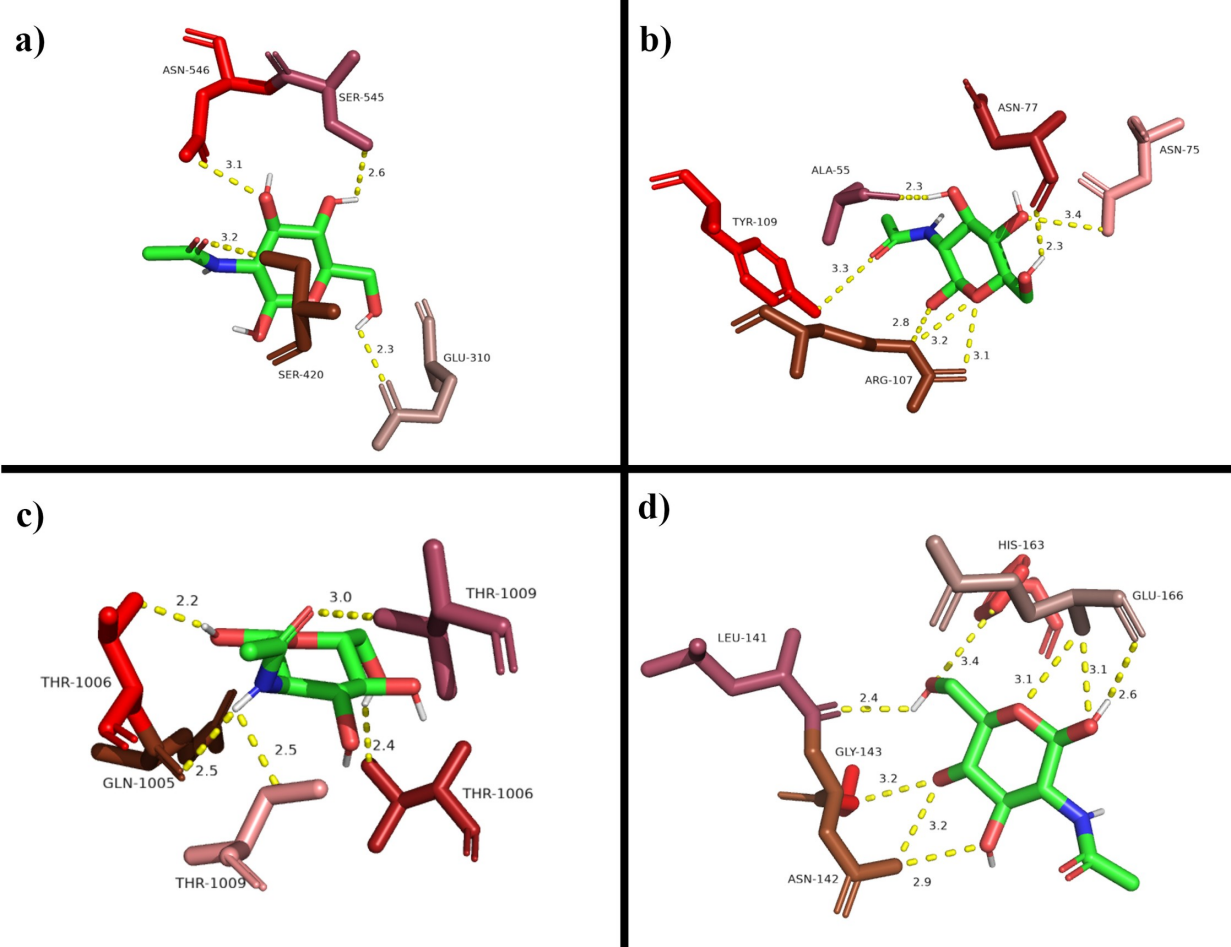
All potential polar interactions between D-GlcNAc (green stick) with a) 6M0J, b) 6WKP, c) 6X79 and d) 7JVZ with 5 Å from D-GlcNAc as predicted by PyMol. The interactions between the residues as shown by yellow dots (length in Å). The amino acid residues are colored randomly for contrast.

The docked structures loaded into PyMol had 5 Å away from the ligand. All the potential polar interactions within this range were highlighted with their measured distances (Figs 3 and 4).

### Molecular dynamics and all analyses

**Fig 5:**
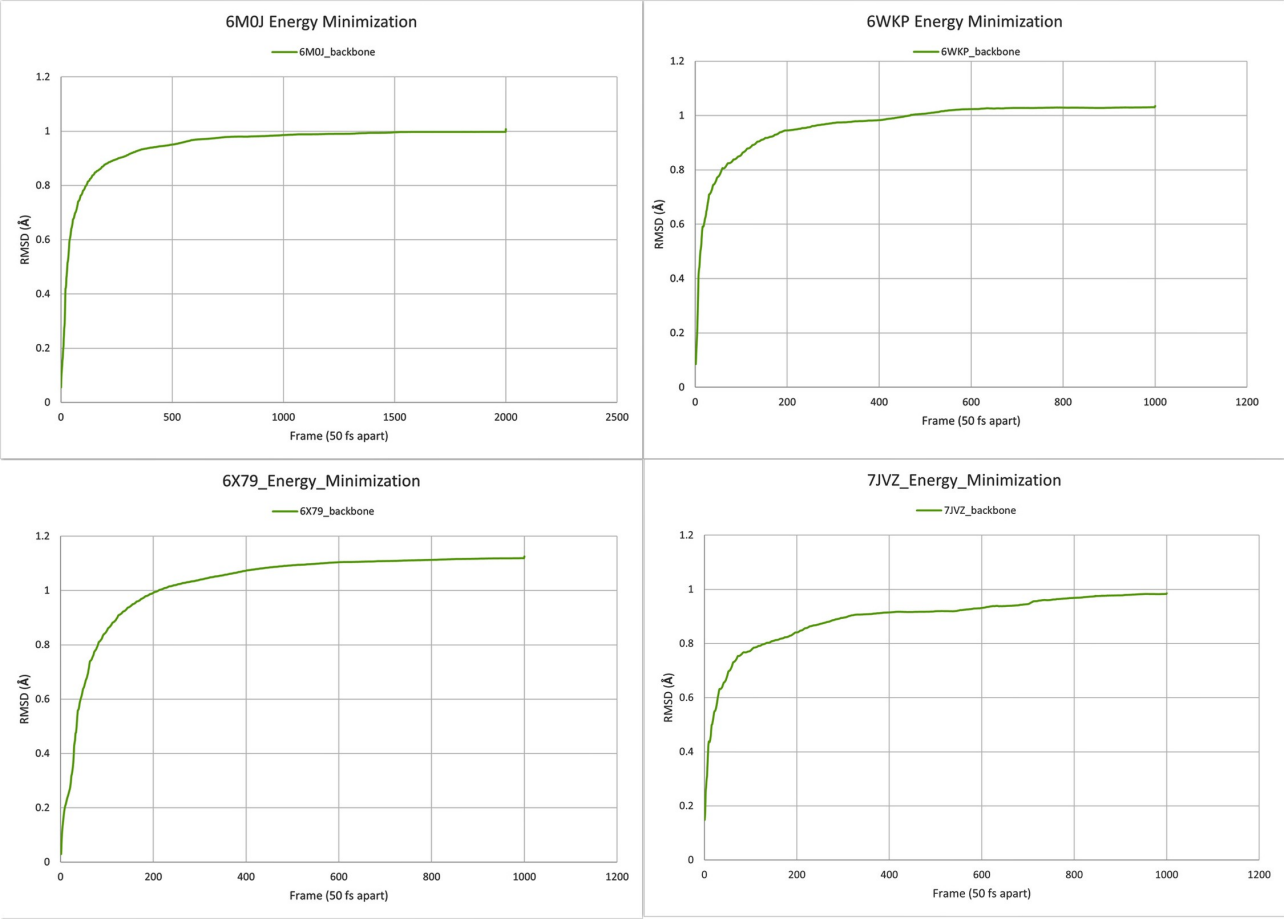
Analysis of minimization trajectory. 100.000 step steepest descent minimization for 6M0J and 50.000 step steepest descent minimization for 6WKP, 6X79, and 7JVZ. Backbone RMSD (Å) along the Y-axis and frames (50 fs distance between any 2 frames) along the X-axis.

**Fig 6:**
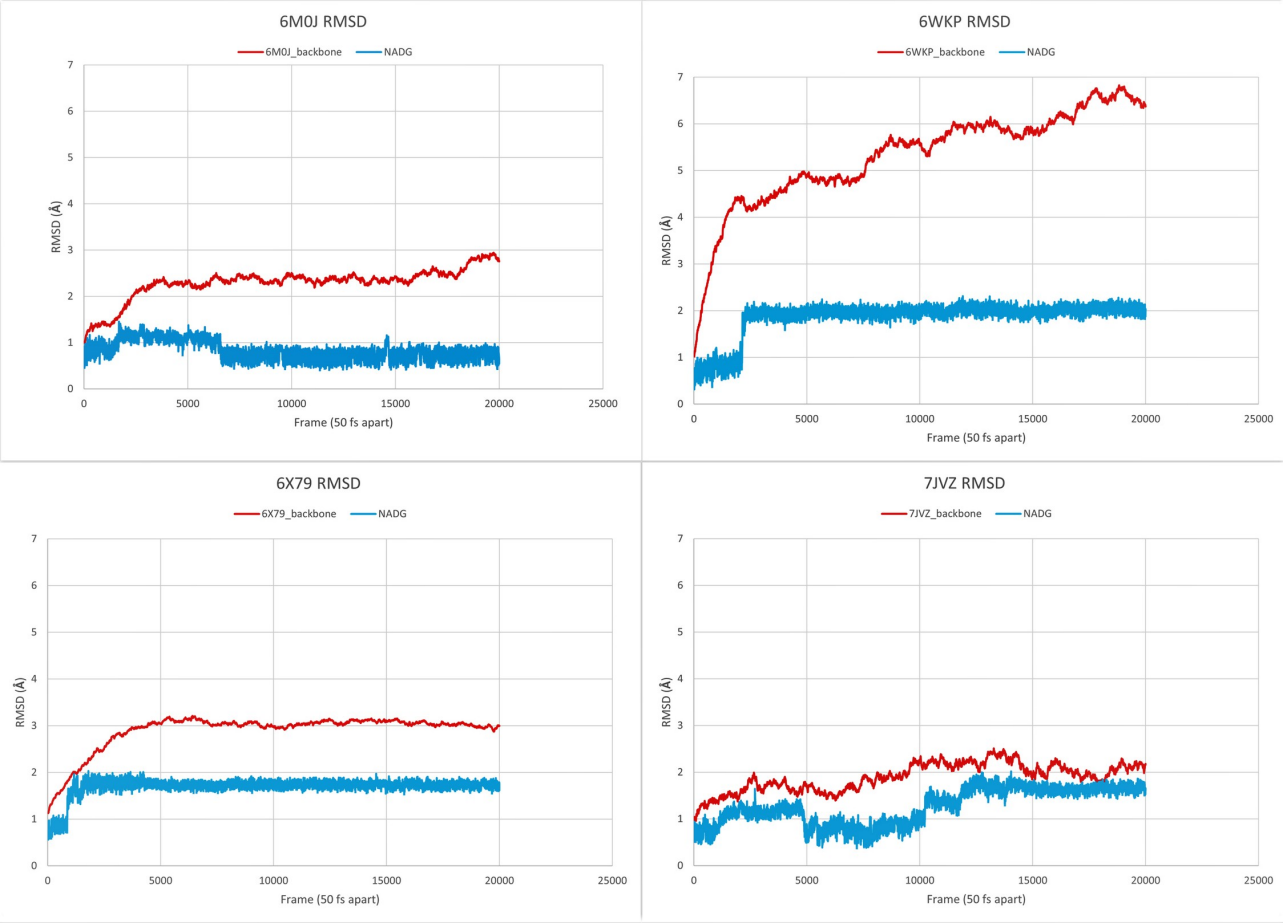
Analysis of molecular dynamics trajectory. Backbone RMSD (Å) along the Y-axis and frames (50 fs distance between any 2 frames) along the X-axis for each protein-ligand complex 1.000.000 production runs. Redline indicating protein backbone RMSD and light blue indicating ligand RMSD.

The RMSD for the backbone (C, Cα, and N) of each protein-ligand complex against their minimization plot was analyzed to check if the minimization steps have successfully minimized the protein-ligand complex, as shown in Fig 5, all the slopes are plateauing near the end frames hence 100.000 steps for 6M0J and 50.000 steps for 6WKP, 6X79 and 7JVZ have produced a minimized structure for the molecular dynamic simulation. Similarly, the backbone RMSD of the protein in protein-ligand complex against their corresponding frames were extracted and plotted, the RMSD of the ligand within the same complex was also plotted in the same graph (Fig 6). The data used for plotting the graphs of both the minimization and production runs are included in S2 Data.

**Fig 7:**
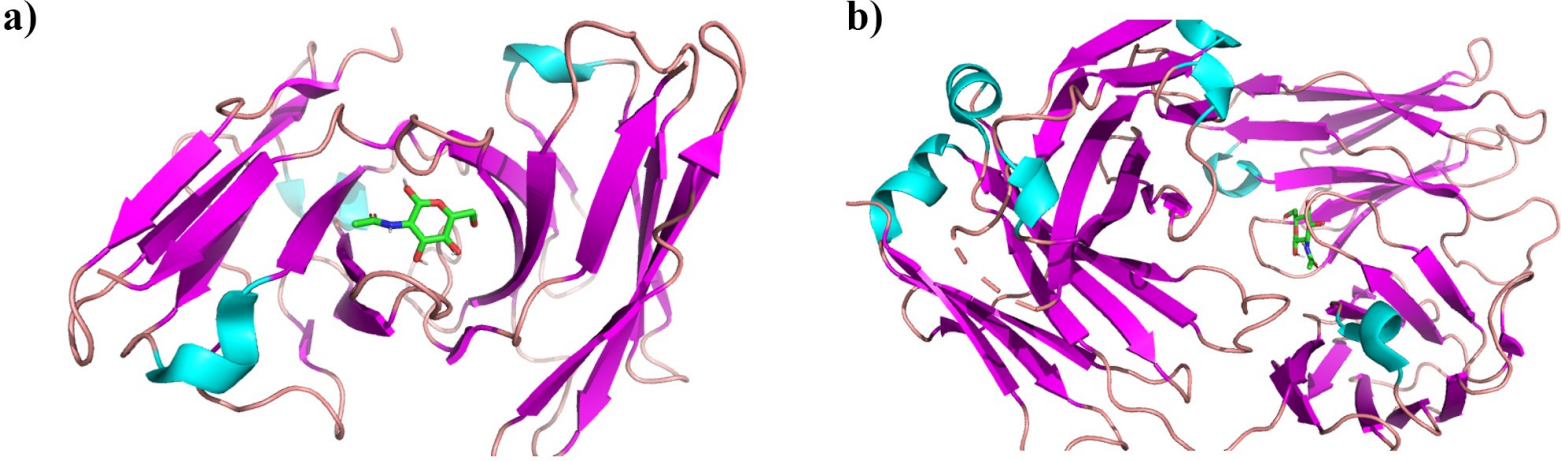
Top docked poses of D-GlcNAc with (a) S2H13 neutralizing antibody Fab fragment from PDB 7JV2 and (b) STAU-281 Fab anti-*Staphylococcus aureus* antibody from PDB 6P9H. All images generated by PyMol, antibody structure represented as cartoons and colored by their secondary structure whereas D-GlcNAc is represented with sticks and colored by element (green).

**Fig 8:**
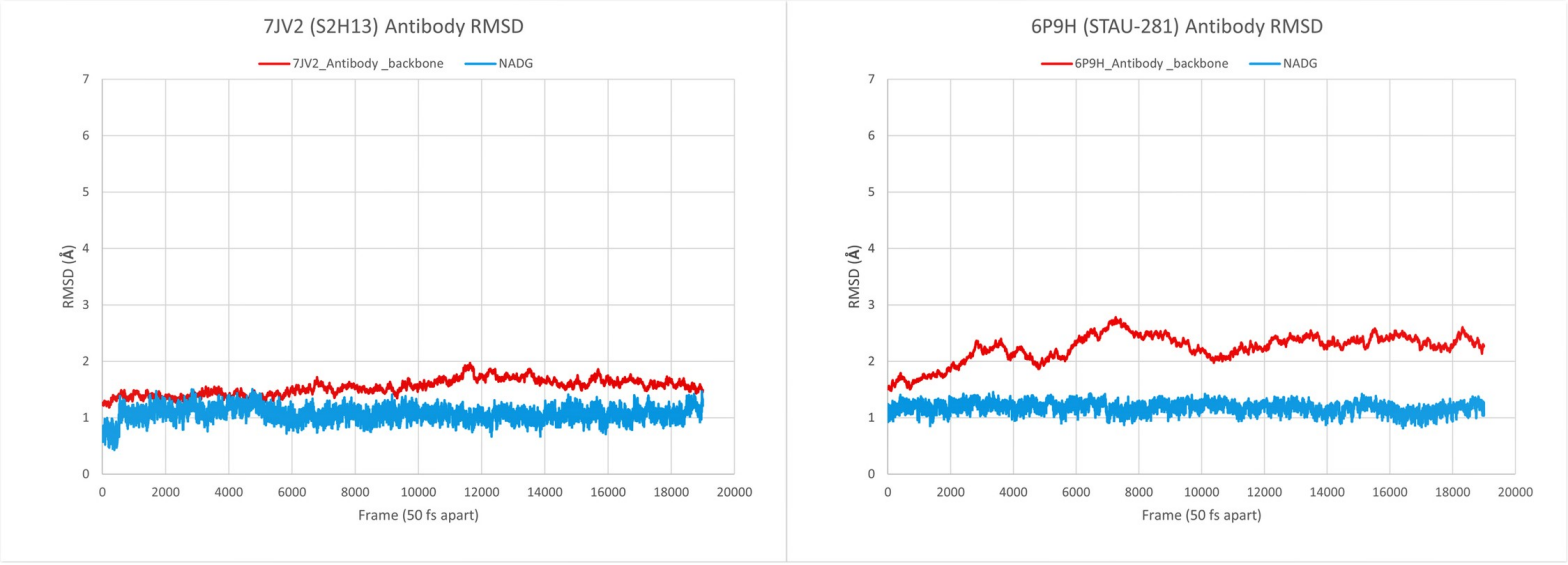
Analysis of molecular dynamics trajectory. Backbone RMSD (Å) along the Y-axis and frames (50 fs distance between any 2 frames) along the X-axis for each protein-ligand complex 1.000.000 production runs. Redline indicating protein backbone RMSD and light blue indicating ligand RMSD.

Both the docking analysis of S2H13 neutralizing antibody Fab fragment and that of anti-*Staphylococcus aureus* antibody (STAU-281 Fab) with D-GlcNAc has shown an affinity of -6.5 kcal/ mol and -6.2 kcal/ mol which satisfies our hypothesis that D-GlcNAc has a significant affinity towards human antibodies (Fig 7) regardless its specificity hence, it has the potential to induce the immune response by bridging a bond between SARS-CoV-2 and antibodies. The RMSD analysis of both the antibodies tested has shown that D-GlcNAc remains at very close proximity to the antibodies (Figs 7 and 8), supporting our hypothesis further. The detailed logfile from the AutoDock Vina and the data used for the RMSD plot are included in S3 and S4 Datas, respectively.

## Discussion

The structural differences considering the active sites of both Mpro proteins cause difficulties in modelling for molecular target. In fact, computational prediction studies carried out involve a massive virtual screening for Mpro inhibitors of SARS-CoV-2 using Deep Docking [33]. The recent studies focused on virtual screening for putative inhibitors of the same main protease of SARS-CoV-2 relied on the clinically approved drugs [34–39] and the compounds according to different databases [40–42]. Molecular docking is a helpful tool to estimate the binding affinity between protein and ligand by using scoring functions [43]. Molecular dynamics simulation as a computational method provides insights into the interaction and atoms according to laws of physics [44].

It is also important to mention the British, South African, and Brazilian variants of SARS-CoV-2 have circulated in the population (as of the time of this article). These variants have shown higher transmissibility due to the collection of few mutations in its spike protein sequence [45–47]. The findings of Cheng and colleagues provided a higher affinity to the human ACE2 receptor, among the mutations, are the N501Y, K417N, and E484K mutations [46]. All these mutations have affected the receptor-binding domain of the spike protein. D-GlcNAc however; as shown in Figs 2 and 3C interacts with the transmembrane domain of the spike protein. These mutations are also irrelevant for the therapeutic potentials of D-GlcNAc we had investigated. Thereby, a molecular docking with the D614G variant of the virus (PDB: 7KDK) and the results did not include any loss of affinity (S5 Data).

We assumed that the loading of D-GlcNAc binding on proteins playing role in pathogenicity and the recognition of SARS-CoV-2 entering host cell through ACE2 receptor side could be increased by the immunologic response. Additionally, D-GlcNAc, which is present in the cell wall of bacteria, has been the reason for the recognition of human IgG1 mAb (F598) through a large groove-shaped binding site cause of the entire light- and heavy-chain interface embedded at least five GlcNAc residues receptor affinity, which makes it possible to recognize the bacterial cells [48,49]. Therefore, it can be suggested that D-GlcNAc can shift immune response, which is responsible for combating with the invading bacteria [49,50], to SARS-CoV-2. In a previous study, D-GlcNAc cluster antigens (TGCA) account for the highest immunoreactivity [51], which could be suggested for the activation of the immune system against SARS-CoV-2. In another study on HIV-1, cause of its structures involving along with glycan-array binding with the variation of the oligosaccharide have shown an existing affinity that affects the neutralizing of antibodies targeting the glycan-shielded trimer on the virus [52]. Correspondingly, this structure and its binding with molecular dynamics could affect the neutralizing of antibodies targeting the virus, we can postulate that this process could change in favor of recognition by antibodies when D-GlcNAc is administrated. This D-GlcNAc stimulated mechanism could also increase the defense capacity of the immunologic response to SARS-CoV-2 invasion. Furthermore, N-acetyl-D-glucosamine-coated polyamidoamine structures induced upregulation of antibody formation on the rat recombinant cells [50] that the similar response could be expected in the human cell.

## Conclusion

The SARS-CoV-2 epidemic has been a major reason threatening human health and resulted in an important economic recession in the world. Our previous findings indicated that D-GlcNAc is a potential compound suggesting strong interaction with proteins involving the ORF1ab region. The data of our previous study showed a drastically unvarying sequence fragment in whole paired genome sequences that can be used as a target origin for designing an effective drug to SARS-CoV-2 [9]. Our results stressed that D-GlcNAc could be considered as a therapeutic option after testing its different concentrations which would be repositioned against SARS-CoV-2.

In the present study, we have carried out molecular dynamics analysis on different proteins of SARS-Cov-2 besides the verification of our previous findings related to binding possibilities with D-GlcNAc and docking analysis. Our results confirmed and highlighted the strong binding of D-GlcNAc to the domain region of the selected proteins on aggressive mutant variants (D614G). We believe that the bacterial cell recognition system could be directed to virus neutralization with the help of D-GlcNAc administration, which results in attaching on both virus and antibodies surface that could increase the epitope binding possibilities via recognition surface, besides the other antibodies. We strongly recommend testing of D-GlcNAc with designed and conducted randomized clinical trials in patients due to its high potential as a repurposed compound.

## Supporting information

S1 Data AutoDock Vina log files for the viral protein docking. (RAR)

S2 Data RMSD data sheet for viral protein molecular dynamics. (RAR)

S3 Data AutoDock Vina log files for antibody docking. (RAR)

S4 Data RMSD data sheet for antibody molecular dynamics. (RAR)

S5 Data Molecular docking of D-GlcNAc with the D614G variant of the virus (PDB: 7KDK). (ZIP)

S1 Graphical abstract (TIF)

## References

[1] Hui DS, Azhar E, Madani TA, Ntoumi F, Kock R, Dar O, et al. The continuing 2019-nCoV epidemic threat of novel coronaviruses to global health—The latest 2019 novel coronavirus outbreak in Wuhan, China. Int J Infect Dis IJID Off Publ Int Soc Infect Dis. 2020; 91:264–6. 10.1016/j.ijid.2020.01.009 31953166 PMC7128332

[2] World Health Organization. WHO Coronavirus (COVID-19) Dashboard; 2021 [cited 2021 May 13]. Database: WHO [Internet]. Available from: https://covid19.who.int.

[3] Guo YR, Cao QD, Hong ZS, Tan YY, Chen SD, Jin HJ, et al. The origin, transmission and clinical therapies on coronavirus disease 2019 (COVID-19) outbreak—an update on the status. Mil Med Res. 2020; 7(1):11. 10.1186/s40779-020-00240-0 32169119 PMC7068984

[4] Phan T. Novel coronavirus: From discovery to clinical diagnostics. Infect Genet Evolut J Mol Epidemiol Evolut Genet Infect Dis. 2020; 79:104211. 10.1016/j.meegid.2020.104211 32007627 PMC7129799

[5] Hoffmann M, Kleine-Weber H, Schroeder S, Kruger N, Herrler T, Erichsen S, et al. SARS-CoV-2 Cell entry depends on ACE2 and TMPRSS2 and is blocked by a clinically proven protease inhibitor. Cell. 2020; 181(2):271–280.e8. 10.1016/j.cell.2020.02.052 32142651 PMC7102627

[6] Yuki K, Fujiogi M, Koutsogiannaki S. COVID-19 pathophysiology: A review. Clin Immunol. 2020; 215:108427. 10.1016/j.clim.2020.108427 32325252 PMC7169933

[7] Denison MR, Graham RL, Donaldson EF, Eckerle LD, Baric RS. Coronaviruses: an RNA proofreading machine regulates replication fidelity and diversity. RNA Biol. 2011; 8(2):270–9. 10.4161/rna.8.2.15013 21593585 PMC3127101

[8] Abdool Karim SS, de Oliveira T. New SARS-CoV-2 variants—clinical, public health, and vaccine implications. N Engl J Med. 2021; 384(19):1866–8. 10.1056/NEJMc2100362 33761203 PMC8008749

[9] Baysal O, Silme RS, Karaaslan C, Ignatov A. Genetic uniformity of a specific region in SARS-CoV-2 genome and repurposing of N-Acetyl-D-Glucosamine. Fresenius Environ Bull. 2021; 30(3):2848–57.

[10] Chen CA, Fang JM. Synthesis of oseltamivir and tamiphosphor from N-acetyl-D-glucosamine. Org Biomol Chem. 2013; 11(44):7687–99. 10.1039/c3ob41622d . 24108094

[11] Chiba S. Effect of early oseltamivir on outpatients without hypoxia with suspected COVID-19. Wien Klin Wochenschr. 2021; 133(7–8):292–7. 10.1007/s00508-020-01780-0 33296027 PMC7724617

[12] Jefferson T, Jones MA, Doshi P, Del Mar CB, Hama R, Thompson MJ, et al. Neuraminidase inhibitors for preventing and treating influenza in adults and children. Cochrane Database Syst Rev. 2014; (4):CD008965. 10.1002/14651858.CD008965.pub4 24718923 PMC6464969

[13] Song N, Qi Q, Cao R, Qin B, Wang B, Wang Y, et al. MAVS O-GlcNAcylation is essential for host antiviral immunity against lethal RNA viruses. Cell Rep. 2019; 28(9):2386–2396.e5 10.1016/j.celrep.2019.07.085 . 31461653

[14] DiNicolantonio JJ, Barroso-Aranda J, McCarty MF. Azithromycin and glucosamine may amplify the type 1 interferon response to RNA viruses in a complementary fashion. Immunol Lett. 2020; 228:83–5. 10.1016/j.imlet.2020.09.008 33002511 PMC7521214

[15] Jourdan JP, Bureau R, Rochais C, Dallemagne P. Drug repositioning: a brief overview. J Pharm Pharmacol. 2020; 72(9):1145–51. 10.1111/jphp.13273 32301512 PMC7262062

[16] Rudrapal M, Khairnar SJ, Jadhav AG. Drug repurposing (DR): an emerging approach in drug discovery. In: Badria FA, editor. Drug repurposing—hypothesis, molecular aspects and therapeutic applications. IntechOpen; 2020. 10.5772/intechopen.93193 Available from: https://www.intechopen.com/books/drug-repurposing-hypothesis-molecular-aspects-and-therapeutic-applications/drug-repurposing-dr-an-emerging-approach-in-drug-discovery.

[17] National Center for Biotechnology Information. PubChem compound summary for CID 439174, N-Acetyl-D-Glucosamine; 2021 [cited 2021 Mar 12]. Database: PubChem [Internet]. Available from: https://pubchem.ncbi.nlm.nih.gov/compound/N-Acetyl-D-Glucosamine.

[18] Lan J, Ge J, Yu J, Shan S, Zhou H, Fan S, et al. Structure of the SARS-CoV-2 spike receptor-binding domain bound to the ACE2 receptor. Nature. 2020; 581(7807):215–20. 10.1038/s41586-020-2180-5 . 32225176

[19] Kang S, Yang M, Hong Z, Zhang L, Huang Z, Chen X, et al. Crystal structure of SARS-CoV-2 nucleocapsid protein RNA binding domain reveals potential unique drug targeting sites. Acta Pharm Sin B. 2020; 10(7):1228–38. 10.1016/j.apsb.2020.04.009 32363136 PMC7194921

[20] McCallum M, Walls AC, Bowen JE, Corti D, Veesler D. Structure-guided covalent stabilization of coronavirus spike glycoprotein trimers in the closed conformation. Nat Struct Mol Biol. 2020; 27(10):942–9. 10.1038/s41594-020-0483-8 32753755 PMC7541350

[21] Schmidt M, Malla T. SARS-CoV-2 Main protease 3CLpro, room temperature, damage free xfel monoclinic structure; 2020 [cited 2021 Mar 12]. Database: RCSB PDB [Internet]. Available from: https://www.rcsb.org/structure/7JVZ 10.2210/pdb7jvz/pdb

[22] Halgren TA. Merck molecular force field. V. Extension of MMFF94 using experimental data, additional computational data, and empirical rules. J Comput Chem. 1996; 17:616–41. 10.1002/(SICI)1096-987X(199604)17:5/6&lt;616::AID-JCC5&gt;3.0.CO;2-X

[23] Pettersen EF, Goddard TD, Huang CC, Couch GS, Greenblatt DM, Meng EC, et al. UCSF Chimera—a visualization system for exploratory research and analysis. J Comput Chem. 2004; 25(13):1605–12. 10.1002/jcc.20084 . 15264254

[24] Morris GM, Huey R, Lindstrom W, Sanner MF, Belew RK, Goodsell DS, et al. AutoDock4 and AutoDockTools4: Automated docking with selective receptor flexibility. J Comput Chem. 2009; 30(16):2785–91. 10.1002/jcc.21256 19399780 PMC2760638

[25] Trott O, Olson AJ. AutoDock Vina: improving the speed and accuracy of docking with a new scoring function, efficient optimization, and multithreading. J Comput Chem. 2010; 31(2):455–61. 10.1002/jcc.21334 19499576 PMC3041641

[26] Dodda LS, Cabeza de Vaca I, Tirado-Rives J, Jorgensen WL. LigParGen web server: an automatic OPLS-AA parameter generator for organic ligands. Nucleic Acids Res. 2017; 45(W1):W331–W336. 10.1093/nar/gkx312 28444340 PMC5793816

[27] Dodda LS, Vilseck JZ, Tirado-Rives J, Jorgensen WL. 1.14*CM1A-LBCC: Localized bond-charge corrected CM1A charges for condensed-phase simulations. J Phys Chem B. 2017; 121(15):3864–70. 10.1021/acs.jpcb.7b00272 28224794 PMC5813481

[28] Jorgensen WL, Tirado-Rives J. Potential energy functions for atomic-level simulations of water and organic and biomolecular systems. Proc Natl Acad Sci. 2005; 102(19):6665–70. 10.1073/pnas.0408037102 15870211 PMC1100738

[29] Humphrey W, Dalke A, Schulten K. VMD: visual molecular dynamics. J Mol Graph. 1996; 14(1):33–8. 10.1016/0263-7855(96)00018-5 . 8744570

[30] Phillips JC, Hardy DJ, Maia JDC, Stone JE, Ribeiro JV, et al. Scalable molecular dynamics on CPU and GPU architectures with NAMD. J Chem Phys. 2020; 153(4):044130. 10.1063/5.0014475 32752662 PMC7395834

[31] Jo S, Kim T, Iyer VG, Im W. CHARMM-GUI: A web-based graphical user interface for CHARMM. J Comput Chem. 2008; 29(11):1859–65. 10.1002/jcc.20945 . 18351591

[32] Schrödinger, LLC. The PyMOL molecular graphics system, version 2.4.1. Available from: https://pymol.org/2/.

[33] Ton A-T, Gentile F, Hsing M, Ban F, Cherkasov A. Rapid identification of potential inhibitors of SARS-CoV-2 main protease by deep docking of 1.3 billion compounds. Mol Inform. 2020; 39(8):2000028. 10.1002/minf.202000028 32162456 PMC7228259

[34] Xu Z, Peng C, Shi Y, Zhu Z, Mu K, Wang X, et al. Nelfinavir was predicted to be a potential inhibitor of 2019-nCov main protease by an integrative approach combining homology modelling, molecular docking and binding free energy calculation. BioRxiv [Preprint]. 2020 bioRxiv 2020.01.27.921627 [posted 2020 Jan 28; cited 2021 Mar 12]: [20 p.]. Available from: https://www.biorxiv.org/content/10.1101/2020.01.27.921627v1

[35] Liu X, Wang X-J. Potential inhibitors against 2019-nCoV coronavirus M protease from clinically approved medicines. J Genet Genomics. 2020; 47(2):119–21. 10.1016/j.jgg.2020.02.001 32173287 PMC7128649

[36] Li Y, Zhang J, Wang N, Li H, Shi Y, Guo G, et al. Therapeutic drugs targeting 2019-nCoV main protease by high-throughput screening. BioRxiv [Preprint]. 2020 bioRxiv 2020.01.28.922922 [posted 2020 Jan 29; revised 2020 Jan 30; cited 2021 Mar 12]: [16 p.]. Available from: https://www.biorxiv.org/content/10.1101/2020.01.28.922922v2

[37] Nguyen DD, Gao K, Chen J, Wang R, Wei G-W. Potentially highly potent drugs for 2019-nCoV. BioRxiv [Preprint]. 2020 bioRxiv 2020.02.05.936013 [posted 2020 Feb 13; cited 2021 Mar 12]: [13 p.]. Available from: https://www.biorxiv.org/content/10.1101/2020.02.05.936013v1 32511344

[38] Talluri S. Molecular docking and virtual screening based prediction of drugs for COVID-19. Comb Chem High Throughput Screen. 2021; 24(5):716–28. 10.2174/1386207323666200814132149 . 32798373

[39] Chen YW, Yiu C-PB, Wong K-Y. Prediction of the SARS-CoV-2 (2019-nCoV) 3C-like protease (3CL (pro)) structure: virtual screening reveals velpatasvir, ledipasvir, and other drug repurposing candidates. F1000Research. 2020; 9:129. 10.12688/f1000research.22457.2 32194944 PMC7062204

[40] Fischer A, Sellner M, Neranjan S, Lill MA, Smieško M. Potential Inhibitors for novel coronavirus protease identified by virtual screening of 606 million compounds. Int J Mol Sci. 2020; 21(10):3626. 10.3390/ijms21103626 32455534 PMC7279339

[41] Gentile D, Patamia V, Scala A, Sciortino MT, Piperno A, Rescifina A. Putative inhibitors of SARS-CoV-2 main protease from a library of marine natural products: a virtual screening and molecular modeling study. Mar Drugs. 2020; 18(4):225. 10.3390/md18040225 32340389 PMC7231030

[42] Adem S, Eyupoglu V, Sarfraz I, Rasul A, Ali M. Identification of potent COVID-19 main protease (Mpro) inhibitors from natural polyphenols: an in silico strategy unveils a hope against CORONA. Preprints [Preprint]. 2020 Preprints 2020030333 [posted 2020 Mar 23; cited 2021 Mar 12]: [16 p.]. Available from: https://www.preprints.org/manuscript/202003.0333/v1 10.20944/preprints202003.0333.v1

[43] Wang G, Zhu W. Molecular docking for drug discovery and development: a widely used approach but far from perfect. Future Med Chem. 2016; 8(14):1707–10. 10.4155/fmc-2016-0143 . 27578269

[44] De Vivo M, Masetti M, Bottegoni G, Cavalli A. Role of molecular dynamics and related methods in drug discovery. J Med Chem. 2016; 59(9):4035–61. 10.1021/acs.jmedchem.5b01684 . 26807648

[45] Conti P, Caraffa A, Gallenga CE, Kritas SK, Frydas I, Younes A, et al. The British variant of the new coronavirus-19 (Sars-Cov-2) should not create a vaccine problem. J Biol Regul Homeost Agents. 2021; 35(1):1–4. 10.23812/21-3-E . 33377359

[46] Cheng MH, Krieger JM, Kaynak B, Arditi M, Bahar I. Impact of South African 501.V2 variant on SARS-CoV-2 spike infectivity and neutralization: a structure-based computational assessment. BioRxiv [Preprint]. 2021 bioRxiv 2021.01.10.426143 [posted 2021 Jan 11; cited 2021 Mar 12]: [7 p.]. Available from: https://www.biorxiv.org/content/10.1101/2021.01.10.426143v1

[47] Dos Santos CA, Bezerra GVB, Azevedo Marinho ARRA, Alves JC, Tanajura DM, Martins-Filho PR. SARS-CoV-2 Genomic surveillance in Northeast Brazil: timing of emergence of the Brazilian variant of concern P1. J Travel Med. 2021; taab066. 10.1093/jtm/taab066 . 33949647 PMC8135834

[48] Soliman C, Walduck AK, Yuriev E, Richards JS, Cywes-Bentley C, Pier GB, et al. Structural basis for antibody targeting of the broadly expressed microbial polysaccharide poly-N-acetylglucosamine. J Biol Chem. 2018; 293(14):5079–89. 10.1074/jbc.RA117.001170 29449370 PMC5892565

[49] Scheffers D-J, Pinho MG. Bacterial cell wall synthesis: new insights from localization studies. Microbiol Mol Biol Rev. 2005; 69(4):585–607. 10.1128/MMBR.69.4.585-607.2005 16339737 PMC1306805

[50] Hulikova K, Benson V, Svoboda J, Sima P, Fiserova A. N-Acetyl-D-glucosamine-coated polyamidoamine dendrimer modulates antibody formation via natural killer cell activation. Int Immunopharmacol. 2009; 9(6):792–9. 10.1016/j.intimp.2009.03.007 . 19303462

[51] Chechik BE, Brockhausen I. Immunochemistry of highly branched N-glycans. Terminal N-acetyl-D-glucosamine (GlcNAc) cluster antigens contain epitopes composed of terminal GlcNAc residues linked to mannose. Biochem Cell Biol. 1988; 66(12):1333–41. 10.1139/o88-154 . 2469444

[52] Stewart-Jones GBE, Soto C, Lemmin T, Chuang G-Y, Druz A, Kong R, et al. Trimeric HIV-1-Env structures define glycan shields from clades A, B, and G. Cell. 2016; 165(4):813–26. 10.1016/j.cell.2016.04.010 27114034 PMC5543418

