# Supplementary figures and images for: Molecular dynamics analysis of N-acetyl-D-glucosamine against specific SARS-CoV-2’s pathogenicity factors
Source: PLoS One. 2021 May 27;16(5):e0252571. doi: 10.1371/journal.pone.0252571 (PMC8158907; doi:10.1371/journal.pone.0252571)

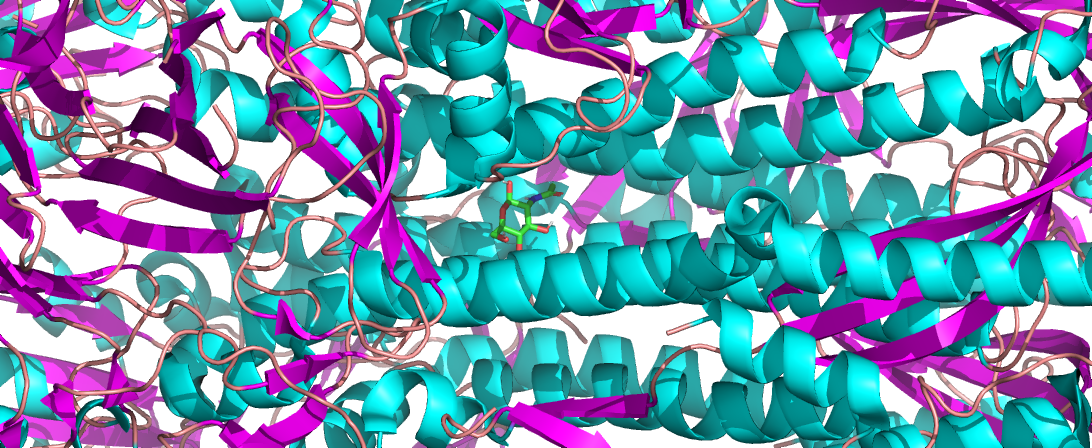

Supplement: S5 Data — (ZIP) [file pone.0252571.s005.zip › 7KDK_docked_opaque.png]

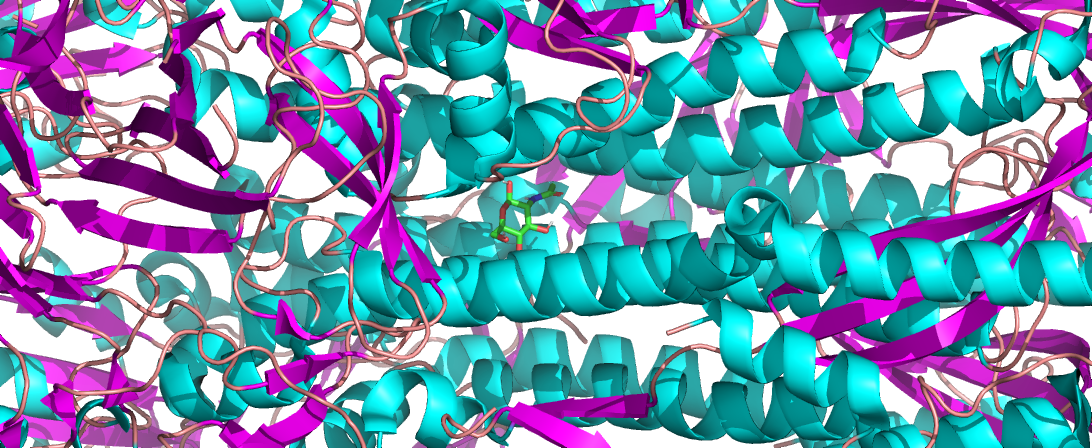

Supplement: S5 Data — (ZIP) [file pone.0252571.s005.zip › 7KDK_docked_transparent.png]

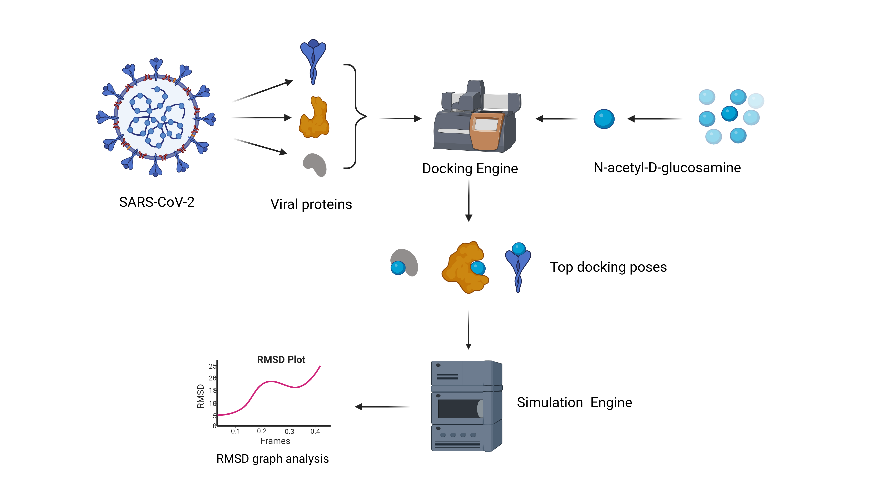

Supplement: S1 Graphical abstract — (TIF) [file pone.0252571.s006.tif]
